# 4-(5-Phenyl-3-trifluoro­meth­yl-1*H*-pyrazol-1-­yl)benzene­sulfonamide

**DOI:** 10.1107/S1600536811033435

**Published:** 2011-08-27

**Authors:** Abdullah M. Asiri, Abdulrahman O. Al-Youbi, Hassan M. Faidallah, Seik Weng Ng, Edward R. T. Tiekink

**Affiliations:** a Chemistry Department, Faculty of Science, King Abdulaziz University, PO Box 80203, Jeddah, Saudi Arabia; b The Center of Excellence for Advanced Materials Research, King Abdulaziz University, Jeddah, PO Box 80203, Saudi Arabia; c Department of Chemistry, University of Malaya, 50603 Kuala Lumpur, Malaysia

## Abstract

Significant twists between the aromatic rings are evident in the structure of the title compound, C_16_H_12_F_3_N_3_O_2_S. With reference to the pyrazole plane, the N- and C-bound benzene rings form dihedral angles of 57.12 (11) and 29.75 (11)°, respectively. The dihedral angle between the benzene rings is 52.82 (11)°. The presence of N—H⋯O(sulfonamide) and N—H⋯N(pyrazole) hydrogen bonds lead to supra­molecular tubes along the *b*-axis direction. These are connected into layers *via* C—H⋯O inter­actions involving a bifurcated O atom (not involved in the N—H⋯O hydrogen bonding). Layers stack along the *a*-axis direction.

## Related literature

For background to the biological applications of related species, see: Faidallah *et al.* (2007 ▶); Al-Saadi *et al.* (2008 ▶). For the crystal structure of a related species, see: Dev *et al.* (1999 ▶).
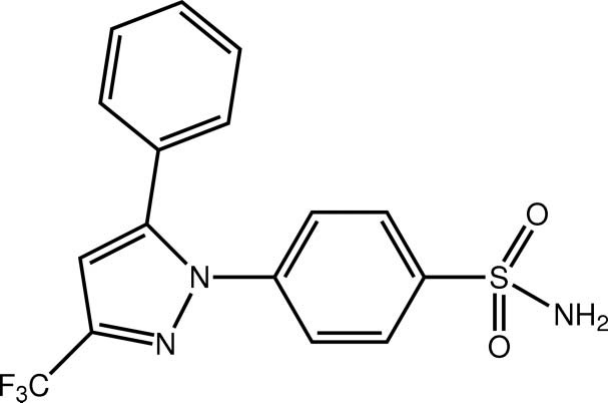


## Experimental

### 

#### Crystal data

C_16_H_12_F_3_N_3_O_2_S
*M* *_r_* = 367.35
Monoclinic, 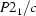

*a* = 16.2430 (7) Å
*b* = 4.9461 (2) Å
*c* = 21.2383 (8) Å
β = 111.231 (5)°
*V* = 1590.47 (11) Å^3^
*Z* = 4
Mo *K*α radiation
μ = 0.25 mm^−1^
*T* = 100 K
0.40 × 0.10 × 0.05 mm

#### Data collection

Agilent SuperNova Dual diffractometer with an Atlas detector
Absorption correction: multi-scan (*CrysAlis PRO*; Agilent, 2010 ▶) *T*_min_ = 0.735, *T*_max_ = 1.000
7901 measured reflections
3560 independent reflections
2876 reflections with *I* > 2σ(*I*)
*R*_int_ = 0.031

#### Refinement

*R*[*F* ^2^ > 2σ(*F* ^2^)] = 0.043
*wR*(*F* ^2^) = 0.113
*S* = 1.06
3560 reflections
234 parameters
H atoms treated by a mixture of independent and constrained refinement
Δρ_max_ = 0.34 e Å^−3^
Δρ_min_ = −0.49 e Å^−3^

### 

Data collection: *CrysAlis PRO* (Agilent, 2010 ▶); cell refinement: *CrysAlis PRO*; data reduction: *CrysAlis PRO*; program(s) used to solve structure: *SHELXS97* (Sheldrick, 2008 ▶); program(s) used to refine structure: *SHELXL97* (Sheldrick, 2008 ▶); molecular graphics: *ORTEP-3* (Farrugia, 1997 ▶) and *DIAMOND* (Brandenburg, 2006 ▶); software used to prepare material for publication: *publCIF* (Westrip, 2010 ▶).

## Supplementary Material

Crystal structure: contains datablock(s) global, I. DOI: 10.1107/S1600536811033435/hg5083sup1.cif

Structure factors: contains datablock(s) I. DOI: 10.1107/S1600536811033435/hg5083Isup2.hkl

Supplementary material file. DOI: 10.1107/S1600536811033435/hg5083Isup3.cml

Additional supplementary materials:  crystallographic information; 3D view; checkCIF report

## Figures and Tables

**Table 1:** Hydrogen-bond geometry (Å, °)

*D*—H⋯*A*	*D*—H	H⋯*A*	*D*⋯*A*	*D*—H⋯*A*
N3—H1⋯O1^i^	0.84 (3)	2.14 (3)	2.911 (2)	153 (2)
N3—H2⋯N2^ii^	0.87 (2)	2.21 (3)	3.049 (3)	164 (2)
C9—H9⋯O2^iii^	0.95	2.49	3.376 (3)	155
C16—H16⋯O2^iv^	0.95	2.55	3.137 (2)	120

Symmetry codes: (i) 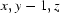; (ii) 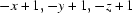; (iii) 
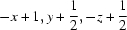
; (iv) 
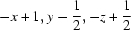
.

**Fig. 1.**
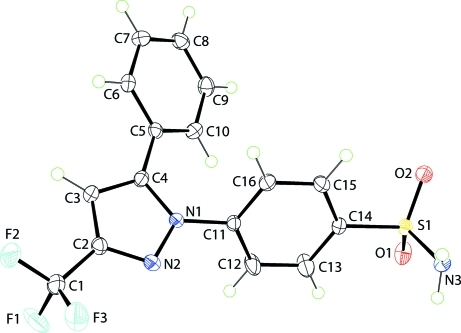
The molecular structure of (I) showing the atom-labelling scheme and displacement ellipsoids at the 50% probability level.

**Fig. 2.**
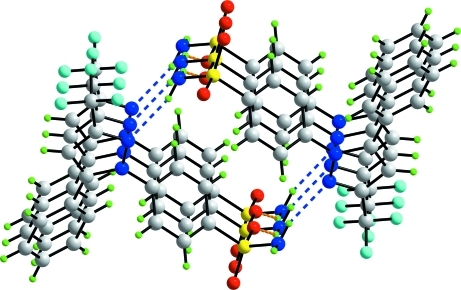
Supramolecular tube aligned along the b axis in (I) mediated by N—H···O and N—H···N hydrogen bonds shown as orange and blue dashed lines, respectively.

**Fig. 3.**
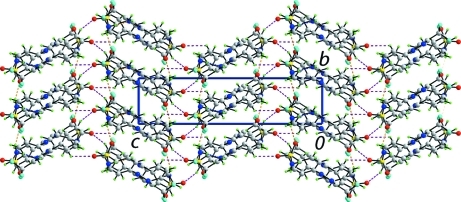
A view in projection down the a axis of the unit-cell contents of (I). The N—H···O and N—H···N hydrogen bonds and C—H···O interactions are shown as orange, blue and purple dashed lines, respectively.

**Fig. 4.**
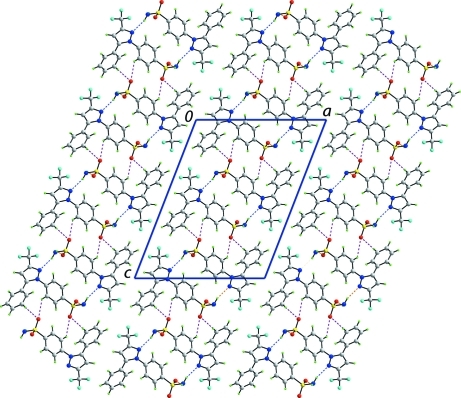
A view in projection down the b axis of the unit-cell contents of (I) highlighting the stacking of layers along the a direction. The N—H···O and N—H···N hydrogen bonds and C—H···O interactions are shown as orange, blue and purple dashed lines, respectively.

## supplementary crystallographic information

### Comment

The crystallographic study of the title compound, (I), which is related to the anti-inflammatory drug, Celecoxib (Dev *et al.*, 1999), was motivated by the recent reports of the biological activities exhibited by related pyrazole compounds (Faidallah *et al.*, 2007; Al-Saadi *et al.*, 2008).

Significant twists are evident in the molecule of (I), Fig. 1. With reference to the pyrazole (N1,N2,C2—C4) plane (r.m.s. deviation = 0,003 Å), the N-bound benzene ring (C11–C16) forms a dihedral angle of 57.12 (11) ° whereas the C-bound benzene ring (C5–C10) forms a dihedral angle of 29.75 (11) °; the dihedral angle formed between the two benzene rings is 52.82 (11) °. The orientations of the benzene rings in (I) differ from those in Celecoxib (Dev *et al.*, 1999), the 4-methylphenyl derivative, with the dihedral angles formed between the N– and C-bound benzene rings and the pyrazole plane being 86.00 (12) and 15.25 (13) °, respectively.

Supramolecular tubes along the *b* axis feature in the crystal packing, Fig. 2. These are stabilized by amino-H hydrogen bonds to the pyrazole-N and to one of the sulfonamide-O atoms, Table 1. Tubes are linked into layers in the *bc* plane by C—H···O interactions whereby the sulfonamide-O2 atom is bifurcated, Table 1 and Fig. 3. Layers stack along the *a* axis, Fig. 4.

### Experimental

A solution of 4,4,4-trifluoro-1-phenyl-1,3-butanedione (2.01 g, 10 mmol) in ethanol (50 ml) was refluxed with 4-hydrazinobenzenesulfonamide hydrochloride (2.2 g, 10 mmol) for 4 h, cooled and diluted with water. The precipitated crude product was filtered and recrystallized from ethanol as colourless crystals; *M*.pt. 431–433 K.

### Refinement

Carbon-bound H-atoms were placed in calculated positions [C—H 0.95 Å, *U*_iso_(H) 1.2*U*_eq_(C)] and were included in the refinement in the riding model approximation. The amino-H atoms were located in a difference Fourier map, and subsequently refined freely.

### Crystal data

**Table:**

C_16_H_12_F_3_N_3_O_2_S	*F*(000) = 752
*M**_r_* = 367.35	*D*_x_ = 1.534 Mg m^−^^3^
Monoclinic, *P*2_1_/*c*	Mo *K*α radiation, λ = 0.71073 Å
Hall symbol: -P 2ybc	Cell parameters from 3338 reflections
*a* = 16.2430 (7) Å	θ = 2.5–29.3°
*b* = 4.9461 (2) Å	µ = 0.25 mm^−^^1^
*c* = 21.2383 (8) Å	*T* = 100 K
β = 111.231 (5)°	Prism, colourless
*V* = 1590.47 (11) Å^3^	0.40 × 0.10 × 0.05 mm
*Z* = 4	

### Data collection

**Table:**

Agilent SuperNova Dual diffractometer with an Atlas detector	3560 independent reflections
Radiation source: SuperNova (Mo) X-ray Source	2876 reflections with *I* > 2σ(*I*)
mirror	*R*_int_ = 0.031
Detector resolution: 10.4041 pixels mm^-1^	θ_max_ = 27.5°, θ_min_ = 2.7°
ω scans	*h* = −16→21
Absorption correction: multi-scan (*CrysAlis PRO*; Agilent, 2010)	*k* = −6→4
*T*_min_ = 0.735, *T*_max_ = 1.000	*l* = −27→27
7901 measured reflections	

### Refinement

**Table:**

Refinement on *F*^2^	Primary atom site location: structure-invariant direct methods
Least-squares matrix: full	Secondary atom site location: difference Fourier map
*R*[*F*^2^ > 2σ(*F*^2^)] = 0.043	Hydrogen site location: inferred from neighbouring sites
*wR*(*F*^2^) = 0.113	H atoms treated by a mixture of independent and constrained refinement
*S* = 1.06	*w* = 1/[σ^2^(*F*_o_^2^) + (0.0453*P*)^2^ + 1.0489*P*] where *P* = (*F*_o_^2^ + 2*F*_c_^2^)/3
3560 reflections	(Δ/σ)_max_ < 0.001
234 parameters	Δρ_max_ = 0.34 e Å^−^^3^
0 restraints	Δρ_min_ = −0.49 e Å^−^^3^

### Special details

**Table:**

Geometry. All e.s.d.'s (except the e.s.d. in the dihedral angle between two l.s. planes) are estimated using the full covariance matrix. The cell e.s.d.'s are taken into account individually in the estimation of e.s.d.'s in distances, angles and torsion angles; correlations between e.s.d.'s in cell parameters are only used when they are defined by crystal symmetry. An approximate (isotropic) treatment of cell e.s.d.'s is used for estimating e.s.d.'s involving l.s. planes.
Refinement. Refinement of *F*^2^ against ALL reflections. The weighted *R*-factor *wR* and goodness of fit *S* are based on *F*^2^, conventional *R*-factors *R* are based on *F*, with *F* set to zero for negative *F*^2^. The threshold expression of *F*^2^ > σ(*F*^2^) is used only for calculating *R*-factors(gt) *etc*. and is not relevant to the choice of reflections for refinement. *R*-factors based on *F*^2^ are statistically about twice as large as those based on *F*, and *R*- factors based on ALL data will be even larger.

### Fractional atomic coordinates and isotropic or equivalent isotropic displacement parameters (Å^2^)

**Table:**

	*x*	*y*	*z*	*U*_iso_*/*U*_eq_	
S1	0.38822 (3)	0.81404 (10)	0.32626 (2)	0.01565 (14)	
F1	0.90203 (11)	0.1680 (3)	0.69086 (6)	0.0425 (4)	
F2	0.94604 (9)	−0.1249 (3)	0.63638 (7)	0.0399 (4)	
F3	0.81737 (9)	−0.1604 (3)	0.64239 (8)	0.0432 (4)	
O1	0.37971 (9)	1.0737 (3)	0.35369 (8)	0.0244 (4)	
O2	0.38267 (9)	0.7931 (3)	0.25777 (7)	0.0231 (3)	
N1	0.74390 (11)	0.3994 (3)	0.49401 (8)	0.0156 (4)	
N2	0.75444 (11)	0.2540 (4)	0.55074 (8)	0.0177 (4)	
N3	0.31318 (11)	0.6258 (4)	0.33572 (9)	0.0164 (4)	
H1	0.3186 (17)	0.461 (6)	0.3280 (12)	0.030 (7)*	
H2	0.3050 (15)	0.658 (5)	0.3732 (12)	0.020 (6)*	
C1	0.87573 (14)	0.0151 (5)	0.63544 (11)	0.0243 (5)	
C2	0.83889 (13)	0.1780 (4)	0.57227 (10)	0.0179 (4)	
C3	0.88288 (13)	0.2711 (4)	0.53112 (10)	0.0183 (4)	
H3	0.9429	0.2415	0.5367	0.022*	
C4	0.82048 (13)	0.4154 (4)	0.48055 (10)	0.0163 (4)	
C5	0.82969 (13)	0.5586 (4)	0.42273 (9)	0.0159 (4)	
C6	0.89089 (13)	0.4628 (4)	0.39583 (10)	0.0195 (4)	
H6	0.9266	0.3111	0.4160	0.023*	
C7	0.90027 (14)	0.5854 (5)	0.34033 (10)	0.0225 (5)	
H7	0.9422	0.5181	0.3226	0.027*	
C8	0.84853 (14)	0.8061 (4)	0.31056 (10)	0.0224 (5)	
H8	0.8542	0.8885	0.2719	0.027*	
C9	0.78859 (14)	0.9066 (4)	0.33714 (11)	0.0229 (5)	
H9	0.7532	1.0587	0.3168	0.027*	
C10	0.77983 (14)	0.7863 (4)	0.39346 (10)	0.0201 (4)	
H10	0.7397	0.8595	0.4122	0.024*	
C11	0.65665 (12)	0.4914 (4)	0.45432 (9)	0.0151 (4)	
C12	0.61316 (15)	0.6612 (5)	0.48304 (11)	0.0324 (6)	
H12	0.6395	0.7133	0.5291	0.039*	
C13	0.52997 (16)	0.7552 (6)	0.44350 (12)	0.0372 (7)	
H13	0.4985	0.8704	0.4627	0.045*	
C14	0.49304 (12)	0.6820 (4)	0.37671 (10)	0.0157 (4)	
C15	0.53662 (14)	0.5093 (4)	0.34853 (10)	0.0207 (5)	
H15	0.5103	0.4580	0.3024	0.025*	
C16	0.61907 (14)	0.4107 (4)	0.38786 (10)	0.0209 (4)	
H16	0.6494	0.2889	0.3692	0.025*	

### Atomic displacement parameters (Å^2^)

**Table:**

	*U*^11^	*U*^22^	*U*^33^	*U*^12^	*U*^13^	*U*^23^
S1	0.0135 (2)	0.0142 (3)	0.0179 (2)	0.00119 (19)	0.00409 (19)	0.00217 (18)
F1	0.0559 (10)	0.0483 (9)	0.0155 (6)	0.0101 (8)	0.0035 (6)	0.0034 (6)
F2	0.0336 (8)	0.0494 (9)	0.0404 (8)	0.0254 (7)	0.0179 (7)	0.0220 (7)
F3	0.0308 (8)	0.0513 (10)	0.0446 (9)	−0.0026 (7)	0.0104 (7)	0.0283 (7)
O1	0.0196 (8)	0.0133 (7)	0.0360 (9)	0.0023 (6)	0.0051 (7)	−0.0020 (6)
O2	0.0185 (7)	0.0320 (9)	0.0189 (7)	0.0016 (7)	0.0068 (6)	0.0080 (6)
N1	0.0152 (8)	0.0185 (9)	0.0130 (8)	0.0028 (7)	0.0050 (6)	0.0008 (7)
N2	0.0190 (9)	0.0194 (9)	0.0143 (8)	0.0009 (7)	0.0056 (7)	0.0017 (7)
N3	0.0170 (9)	0.0144 (9)	0.0187 (9)	0.0009 (7)	0.0075 (7)	−0.0001 (7)
C1	0.0202 (11)	0.0282 (12)	0.0247 (11)	0.0059 (9)	0.0083 (9)	0.0065 (9)
C2	0.0174 (10)	0.0186 (10)	0.0167 (9)	0.0034 (8)	0.0048 (8)	0.0006 (8)
C3	0.0147 (10)	0.0214 (10)	0.0187 (10)	0.0024 (8)	0.0061 (8)	−0.0001 (8)
C4	0.0136 (9)	0.0174 (10)	0.0175 (9)	0.0005 (8)	0.0053 (8)	−0.0026 (8)
C5	0.0137 (9)	0.0180 (10)	0.0145 (9)	−0.0032 (8)	0.0034 (7)	−0.0013 (8)
C6	0.0150 (10)	0.0226 (11)	0.0195 (10)	0.0024 (8)	0.0043 (8)	0.0015 (8)
C7	0.0224 (11)	0.0276 (12)	0.0201 (10)	0.0000 (9)	0.0110 (9)	−0.0006 (9)
C8	0.0234 (11)	0.0266 (11)	0.0173 (10)	−0.0043 (9)	0.0073 (9)	0.0025 (9)
C9	0.0213 (11)	0.0205 (11)	0.0243 (11)	0.0018 (9)	0.0052 (9)	0.0048 (9)
C10	0.0191 (10)	0.0199 (11)	0.0231 (10)	0.0010 (9)	0.0099 (9)	−0.0015 (9)
C11	0.0129 (9)	0.0166 (10)	0.0152 (9)	0.0010 (8)	0.0043 (8)	0.0025 (8)
C12	0.0244 (12)	0.0470 (15)	0.0183 (10)	0.0133 (11)	−0.0013 (9)	−0.0148 (10)
C13	0.0272 (13)	0.0526 (16)	0.0243 (12)	0.0208 (12)	0.0003 (10)	−0.0182 (11)
C14	0.0134 (9)	0.0151 (10)	0.0176 (9)	0.0004 (8)	0.0046 (8)	0.0013 (8)
C15	0.0191 (10)	0.0263 (11)	0.0151 (9)	0.0022 (9)	0.0045 (8)	−0.0036 (9)
C16	0.0185 (10)	0.0249 (11)	0.0194 (10)	0.0065 (9)	0.0070 (8)	−0.0034 (9)

### Geometric parameters (Å, °)

**Table:**

S1—O2	1.4283 (15)	C6—C7	1.382 (3)
S1—O1	1.4373 (15)	C6—H6	0.9500
S1—N3	1.6033 (18)	C7—C8	1.383 (3)
S1—C14	1.7754 (19)	C7—H7	0.9500
F1—C1	1.332 (3)	C8—C9	1.383 (3)
F2—C1	1.330 (2)	C8—H8	0.9500
F3—C1	1.332 (3)	C9—C10	1.389 (3)
N1—N2	1.359 (2)	C9—H9	0.9500
N1—C4	1.376 (3)	C10—H10	0.9500
N1—C11	1.436 (2)	C11—C12	1.374 (3)
N2—C2	1.333 (3)	C11—C16	1.378 (3)
N3—H1	0.84 (3)	C12—C13	1.387 (3)
N3—H2	0.87 (2)	C12—H12	0.9500
C1—C2	1.492 (3)	C13—C14	1.374 (3)
C2—C3	1.392 (3)	C13—H13	0.9500
C3—C4	1.379 (3)	C14—C15	1.377 (3)
C3—H3	0.9500	C15—C16	1.385 (3)
C4—C5	1.471 (3)	C15—H15	0.9500
C5—C6	1.397 (3)	C16—H16	0.9500
C5—C10	1.395 (3)		
			
O2—S1—O1	119.85 (9)	C7—C6—H6	119.5
O2—S1—N3	108.36 (9)	C5—C6—H6	119.5
O1—S1—N3	106.20 (10)	C6—C7—C8	120.0 (2)
O2—S1—C14	106.32 (9)	C6—C7—H7	120.0
O1—S1—C14	107.22 (9)	C8—C7—H7	120.0
N3—S1—C14	108.50 (9)	C9—C8—C7	119.9 (2)
N2—N1—C4	112.43 (16)	C9—C8—H8	120.1
N2—N1—C11	117.95 (15)	C7—C8—H8	120.1
C4—N1—C11	129.36 (16)	C8—C9—C10	120.3 (2)
C2—N2—N1	103.70 (16)	C8—C9—H9	119.9
S1—N3—H1	113.6 (18)	C10—C9—H9	119.9
S1—N3—H2	112.6 (16)	C9—C10—C5	120.4 (2)
H1—N3—H2	115 (2)	C9—C10—H10	119.8
F2—C1—F1	106.47 (18)	C5—C10—H10	119.8
F2—C1—F3	107.48 (19)	C12—C11—C16	121.55 (19)
F1—C1—F3	106.57 (18)	C12—C11—N1	119.34 (17)
F2—C1—C2	110.94 (18)	C16—C11—N1	119.11 (18)
F1—C1—C2	112.57 (19)	C11—C12—C13	118.7 (2)
F3—C1—C2	112.45 (18)	C11—C12—H12	120.6
N2—C2—C3	112.93 (18)	C13—C12—H12	120.6
N2—C2—C1	119.24 (18)	C14—C13—C12	120.1 (2)
C3—C2—C1	127.82 (19)	C14—C13—H13	119.9
C4—C3—C2	105.10 (18)	C12—C13—H13	119.9
C4—C3—H3	127.5	C13—C14—C15	120.76 (19)
C2—C3—H3	127.5	C13—C14—S1	119.41 (16)
N1—C4—C3	105.85 (17)	C15—C14—S1	119.83 (15)
N1—C4—C5	125.03 (17)	C14—C15—C16	119.55 (19)
C3—C4—C5	129.12 (18)	C14—C15—H15	120.2
C6—C5—C10	118.42 (19)	C16—C15—H15	120.2
C6—C5—C4	118.74 (18)	C11—C16—C15	119.24 (19)
C10—C5—C4	122.85 (18)	C11—C16—H16	120.4
C7—C6—C5	121.0 (2)	C15—C16—H16	120.4
			
C4—N1—N2—C2	−0.4 (2)	C6—C7—C8—C9	1.1 (3)
C11—N1—N2—C2	174.33 (17)	C7—C8—C9—C10	−0.3 (3)
N1—N2—C2—C3	0.2 (2)	C8—C9—C10—C5	−1.7 (3)
N1—N2—C2—C1	179.01 (18)	C6—C5—C10—C9	2.7 (3)
F2—C1—C2—N2	156.75 (19)	C4—C5—C10—C9	−177.26 (19)
F1—C1—C2—N2	−84.1 (2)	N2—N1—C11—C12	60.5 (3)
F3—C1—C2—N2	36.3 (3)	C4—N1—C11—C12	−125.9 (2)
F2—C1—C2—C3	−24.6 (3)	N2—N1—C11—C16	−120.6 (2)
F1—C1—C2—C3	94.6 (3)	C4—N1—C11—C16	53.0 (3)
F3—C1—C2—C3	−145.0 (2)	C16—C11—C12—C13	−0.8 (4)
N2—C2—C3—C4	0.1 (2)	N1—C11—C12—C13	178.1 (2)
C1—C2—C3—C4	−178.6 (2)	C11—C12—C13—C14	−1.0 (4)
N2—N1—C4—C3	0.4 (2)	C12—C13—C14—C15	1.8 (4)
C11—N1—C4—C3	−173.52 (19)	C12—C13—C14—S1	−177.8 (2)
N2—N1—C4—C5	179.93 (17)	O2—S1—C14—C13	158.1 (2)
C11—N1—C4—C5	6.0 (3)	O1—S1—C14—C13	28.8 (2)
C2—C3—C4—N1	−0.3 (2)	N3—S1—C14—C13	−85.5 (2)
C2—C3—C4—C5	−179.8 (2)	O2—S1—C14—C15	−21.5 (2)
N1—C4—C5—C6	−149.8 (2)	O1—S1—C14—C15	−150.81 (17)
C3—C4—C5—C6	29.6 (3)	N3—S1—C14—C15	94.88 (19)
N1—C4—C5—C10	30.2 (3)	C13—C14—C15—C16	−0.7 (3)
C3—C4—C5—C10	−150.4 (2)	S1—C14—C15—C16	178.87 (16)
C10—C5—C6—C7	−1.8 (3)	C12—C11—C16—C15	1.9 (3)
C4—C5—C6—C7	178.14 (19)	N1—C11—C16—C15	−176.98 (18)
C5—C6—C7—C8	−0.1 (3)	C14—C15—C16—C11	−1.1 (3)

### Hydrogen-bond geometry (Å, °)

**Table:**

*D*—H···*A*	*D*—H	H···*A*	*D*···*A*	*D*—H···*A*
N3—H1···O1^i^	0.84 (3)	2.14 (3)	2.911 (2)	153 (2)
N3—H2···N2^ii^	0.87 (2)	2.21 (3)	3.049 (3)	164 (2)
C9—H9···O2^iii^	0.95	2.49	3.376 (3)	155
C16—H16···O2^iv^	0.95	2.55	3.137 (2)	120

Symmetry codes: (i) *x*, *y*−1, *z*; (ii) −*x*+1, −*y*+1, −*z*+1; (iii) −*x*+1, *y*+1/2, −*z*+1/2; (iv) −*x*+1, *y*−1/2, −*z*+1/2.
